# Long Term Variceal Sclerotherapy: Is Endoscopic Sclerosis a Unique Therapeutic Approach and a True Alternative to Surgery?

**DOI:** 10.1155/1991/32191

**Published:** 1991

**Authors:** K-J. Paquet, A. Lazar, W. Rambach

**Affiliations:** Department of Surgery and Medicine HEINZ-KALK-Hospital Bad Kissingen D-8730 Germany

## Abstract

Endoscopic sclerotherapy has been used to control acute variceal haemorrhage which persists despite conservative therapy, prevent recurrent variceal haemorrhage in patients with a history of oesophageal haemorrhage, and to prevent a haemorrhage in patients with oesophageal varices who never bled.

In this short paper I will cover our personal experience with more than 2000 patients receiving particularly paravariceal endoscopic sclerotherapy of bleeding esophageal varices, and especially present the results of our prospective and controlled randomized trials (Table 1) and underline the thesis that endoscopic sclerotherapy and surgical procedures for patients with portal hypertension are complementary supporting measures or options.


*HPB Surgery*, 1991. Vol. 4, pp 11–25
Reprints available directly from the publisher
Photocopying permitted by license only

© 1991 Harwood Academic Publishers GmbH
Printed in the United Kingdom

### LONG TERM VARICEAL SCLEROTHERAPY: IS ENDOSCOPIC SCLEROSIS A UNIQUE THERAPEUTIC APPROACH AND A TRUE ALTERNATIVE TO SURGERY?

K-J. PAQUET, A. LAZAR, W. RAMBACH
*Department of Surgery and Medicine, HEINZ-KALK-Hospital, D–8730 Bad Kissingen, Germany*

*(Received 18 September 1990)*

Endoscopic sclerotherapy has been used to control acute variceal haemorrhage which persists despite conservative therapy, prevent recurrent variceal haemorrhage in patients with a history of oesophageal haemorrhage, and to prevent a haemorrhage in patients with oesophageal varices who never bled.

In this short paper I will cover our personal experience with more than 2000 patients receiving particularly paravariceal endoscopic sclerotherapy of bleeding esophageal varices, and especially present the results of our prospective and controlled randomized trials (Table 1) and underline the thesis that endoscopic sclerotherapy and surgical procedures for patients with portal hypertension are complementary supporting measures or options.

KEY WORDS: Variceal sclerotherapy, surgical procedures, bleeding esophageal varices

**Table 1** Different groups with portal hypertension (95% cirrhotics) treated by mainly paravariceal endoscopic sclerotherapy Group I–Group IV

| | |
|---|---|
| Group I : | Acute an uncontrollable variceal haemorrhage n = 653<br>– 22 (Group Ia)–232 (Group Ib) = 399 |
| Group Ia: | Acute variceal haemorrhage – prospective randomized controlled trial<br>(n = 22 (43)) Jan. 1, 1980 – Jan. 1 1983 |
| Group Ib: | Prospective evaluation<br>(n = 232) Jan. 1, 1982 – Jan. 1, 1987 |
| Group II : | Elective treatment of variceal haemorrhage (n = 1247) |
| Group IIIa: | Prospective treatment of oesophageal varices (n = 36 (72))<br>– *First* prospective randomized controlled trial<br>(Jan. 1, 1978 – Jan. 1, 1980) |
| Group IIIb: | Prophylactic treatment of oesophageal varices (n = 43 (85))<br>– *Second* prospective randomized ongoing trial (Sept. 1, 1987 – *July 1, 1990*) |
| Group IV : | Acute and elective treatment of variceal haemorrhage in babies and children (n = 71) |

Correspondence to: K.-J. Paquet, Department of Surgery, HEINZ-KALK-Hospital, D–8730 Bad Kissingen, Germany

### INITIAL MANAGEMENT

Emergency endoscopic sclerotherapy can be performed immediately, at the time of the first diagnostic endoscopy, as preferred and recommended by our group or it can be delayed until the variceal haemorrhage has been controlled by conservative measures with or without the use of a pharmacologic agent or balloon tamponade. The use of immediate sclerotherapy requires a high degree of skill. We recommend its use whenever possible, since it provides instant control of haemorrhage. If these conditions are not fullfilled, we recommend pharmacological therapy or balloon tamponade and transfer of the patient to a specialist center.

**Table 2** Results of controlled trials of emergency injection sclerotherapy of bleeding esophageal varices

| *Reference* | *No. of patients* | *Method of emergency injection sclerotherapy i.v., p.v.* | *Haemostasis (immediate) (%) (sc/c)* | *Survival rate after 1 year (%)* |
|---|---|---|---|---|
| Paquet and Feussner 1985 | 21 | Polidocanol 0.5 + 1% p.v. | 90 (55) | 79 (38) |
| Larson *et al.* 1986 | 44 | Tetradecylsulfate 3% i.v. | 85 (47) | 62 (54) |

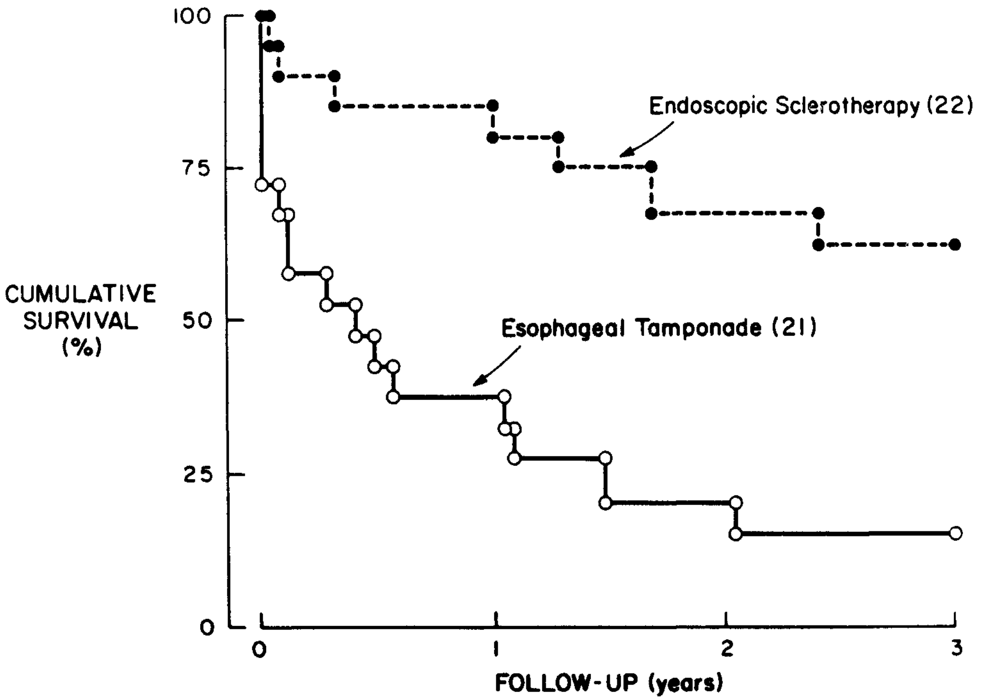

**Figure 1** Cumulative survival curve using the method of KAPLAN-MEIER for the controlled randomized trial comparing the SENGSTAKEN-BLAKEMORE tube with emergency endoscopic sclerotherapy during emergency.

**Table 3** Aetiology of the intra- and prehepatic block of prospective evaluation of immediate endoscopic injection sclerosis (IES) (n = 232; Jan. 1, 1982 – Jan. 1, 1987)

| | *Number* | *Percent* |
|---|---|---|
| *A. Underlying disease* | | |
| Alcoholic cirrhosis | 138 | 59.5 |
| Posthepatitic cirrhosis | 47 | 20.3 |
| Cirrhosis of unknown aetiology | 17 | 7.3 |
| Primary biliary cirrhosis | 11 | 4.7 |
| Extrahepatic bile duct atresia | 2 | 0.9 |
| Secondary biliary cirrhosis | 1 | 0.4 |
| Liver cirrhosis (total) | 216 | 93.1 |
| Prehepatic block | 9 | 3.9 |
| Liver fibrosis | 5 | 2.2 |
| Schistosomiasis | 1 | 0.4 |
| Mucoviscidosis | 1 | 0.4 |
| Non-cirrhotic patients (total) | 16 | 6.9 |
| *B. Classification* | | |
| CHILD-PUGH A* | 53 | 23 |
| CHILD-PUGH B | 70 | 30 |
| CHILD-PUGH C | 109 | 47 |
| Total | 232 | 100 |

* Non-cirrhotic patients are classified as CHILD-PUGH A

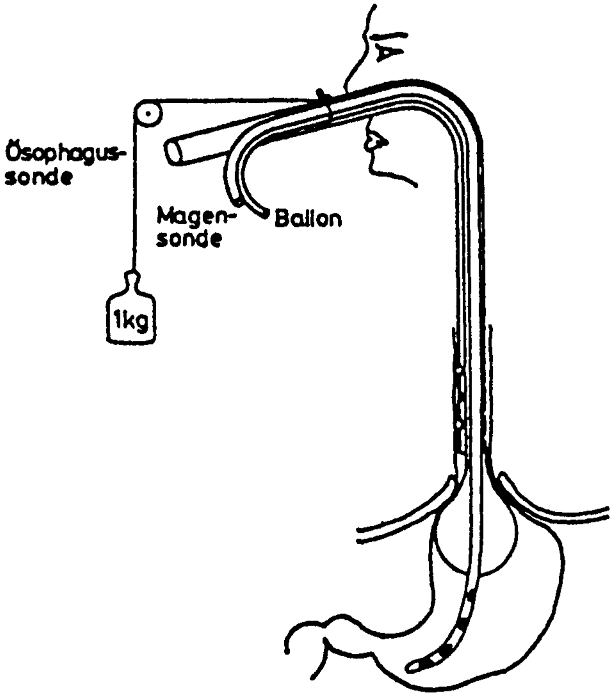

**Figure 2** Schematic presentation of the use of the LINTON-NACHLAS tube.

After sclerotherapy has controlled the haemorrhage, we support the view that at least two to four additional sessions are necessary to obliterate the varices by intravariceal or combined injection or to protect them by scar tissue by paravariceal injections. In two controlled trials — one by our group — it was demonstrated that emergency injection sclerotherapy significantly improved haemostastis and survival in comparison with other conservative measures (Table 2)[1,2].

The cumulative survival curve using the method of KAPLAN-MEIER (Figure 1) demonstrates a statistically significant difference in favour of sclerotherapy (IEIS) after six months ($p < 0.05$) and a higher significance after 36 months ($p < 0.0005$). We prospectively treated 232 patients from January 1, 1982 to January 1, 1987 with the following CHILD-PUGH criteria[3]: 53 (23%) A, 70 (30%) B and 109 (47%) C. More than 93% had liver cirrhosis, with 60% being of alcoholic origin (Table 3). If IEIS by the free hand technique was not successful after 15 minutes a LINTON-NACHLAS tube (Figure 2) was inserted for 6–12 hours. In cases of

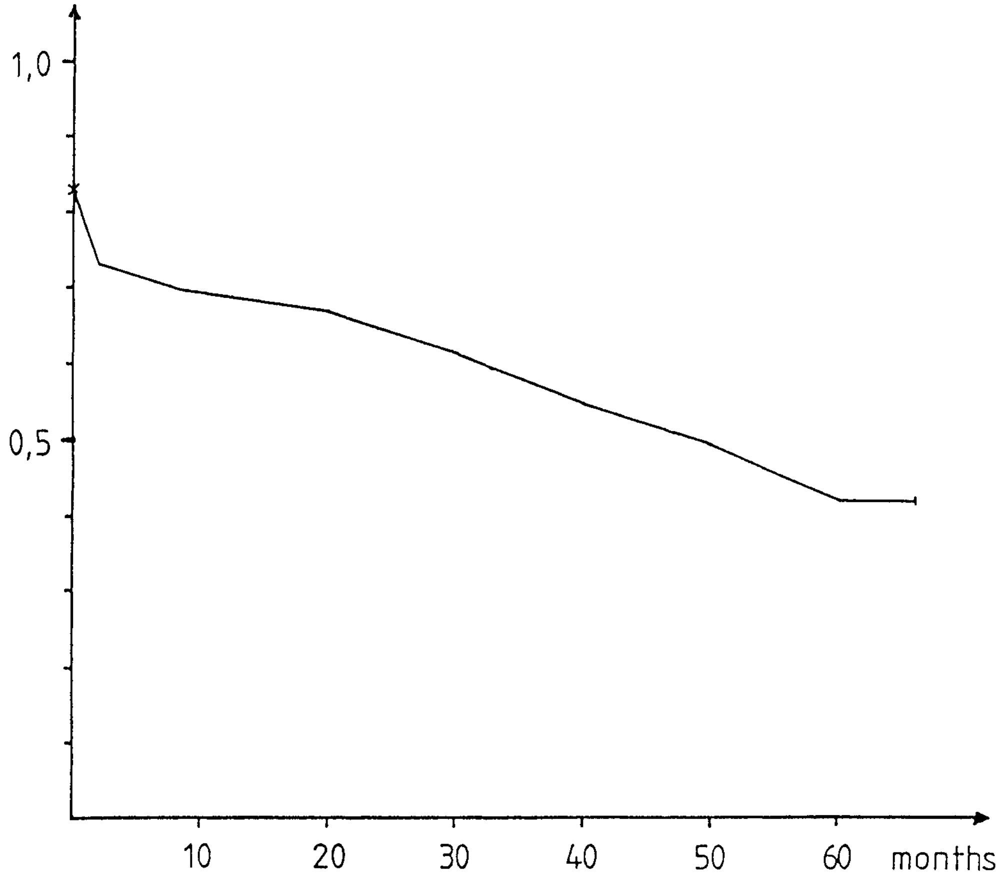

**Figure 3** Cumulatic survival curve after immediate endoscopic injection sclerotherapy of bleeding oesophageal varices during emergency endoscopy (in the trial surgical treatment after sclerotherapy failure is included).

recurrence of haemorrhage a second emergency endoscopy and IEIS and, if this was not successful, a gastroesophageal disconnection (look Fig. 7) was performed directly. During the bleeding free interval CHILD-PUGH A- and -B-patients were selected using special criteria for shunt operation. All sclerotherapy patients were checked after 4 months and thereafter every 6, 9 and 12 months and reinjected if necessary. Bleeding was controlled in 93% with IEIS and in 97% with the combination of IEIS and LINTON-NACHLAS tube. Definite control of haemorrhage was accomplished in 94%. Thirty-five patients died during the first days of admission (15.1%). The main causes of death were liver failure and variceal haemorrhage. Only 2 patients were lost to follow up. The main causes of 39 late deaths (29.8%) were liver failure, hepatocellular cancer and haemorrhage. The calculated cumulative survival curve using the method of KAPLAN-MEIER demonstrates a five-year life expectancy of about 50% (Figure 3). As fixed in the protocol of the trial, surgery as treatment option was included in case of persistant and/or recurrent variceal haemorrhage in spite of effective sclerotherapy. Number, type and mortality or surgical procedure are listed in Table 4: mortality after emergency gastroesophageal disconnection (19 cases) was 31.6 and after selective and non-selective shunt procedures, all performed within the two weeks after successful endoscopic control of recurrent variceal haemorrhage was 11.4%.

**Table 4** Number, type and mortality of surgical procedures due to persistant and/or recurrent variceal haemorrhage in spite of effective sclerotherapy

| | | *Mortality* | |
|---|---|---|---|
| *Number* | *Type* | *Number* | *Percent* |
| 19 | Gastro-oesophageal disconnection according to HASSAB-PAQUET | 6 | 31.6 |
| 35 | Selective and non-selective shunts | 4 | 11.4 |
| 18 | Meso-caval interposition | 2 | 11.1 |
| 16 | Spleno-renal (WARREN) | 2 | 12.5 |
| 1 | Porto-caval (end-to-side) | 0 | 0 |
| 54 (24%) | | 10 | 18.5 |

Thus, IEIS during emergency endoscopy is established as a primary therapeutic mode to successfully control bleeding oesophageal varices. It appears to be superior to elective sclerotherapy. In spite of that we recommend this strategy only for a very experienced operator and endoscopist who must be available day and night and who also has an experienced endoscopy team with at least two additional persons to hand.

### *Elective Endoscopic Sclerotherapy*

Although repeated injection sclerotherapy is the most widely practised long-term treatment after variceal bleeding, it has not yet been proved to be the single most effective form of management. Five major controlled trials (Table 5) have evaluated long-term endoscopic sclerotherapy for the prevention of rebleeding[4–8]. In all these studies the frequency of rebleeding was reduced in sclerotherapy patients, although in three trials the difference to the control group was only significant when the total number of the bleeding episodes were considered rather than the number of patients who bled. However, up to 50% of patients rebleed on chronic sclerotherapy, although most of the rebleeding occurs before complete eradication or protection by scar tissue of the varices has been achieved and is of minor severity. The beneficial effect of long-term sclerotherapy on survival, as convincingly demonstrated in the trial from the Kings College Hospital[4] in London (Figure 4) and confirmed by our group (Tables 6, 7, 8) is nevertheless debated. Rebleeding in the control group of one trial was routinely treated by acute sclerosis. By the end of this trial, most control patients had received at least one session of sclerotherapy[5]. Thus, similar survival in both groups is not surprising (Figure 5). The Copenhagen

**Table 5** The effect of elective endoscopic sclerotherapy on the incidence of rebleeding and survival in randomized controlled trials of patients with variceal bleeding and cirrhosis of the liver (sc = sclerotherapy; c = control)*

| *Reference* | *No. of patients* (*sc*/c) | *Rebleeding rate after 1 year (%)* (*sc*/c) | *Survival 2 years (%)* (*sc*/c) |
|---|---|---|---|
| Westaby, MacDougall *et al.* (1983) | *56/60* | *49/79* (significantly different) p<0.01 | *78/43* (significantly different) p<0.01 |
| Terblance *et al.* (1983) | *38/37* | *67/82* (significantly different) p<0.02 | *45/45* (no difference) |
| Copenhagen-ES-Trial (1984) | *93/94* | *31/60* (significantly different from the 40th day) p<0.01 | *78/65* (significantly different after 40 days) p<0.05 |
| Söderlund (1985) | *54/53* | *46/66* (significantly different) p<0.05 | *82/58* (significantly different) p<0.01 |
| Korula *et al.* (1985) | *56/60* | *47/71* (significantly different) p<0.01 | *51/35* (significantly different only if urgent shunts are excluded) |

* The percentages were calculated by adding the number of individuals who had rebled or survived, and dividing this by the total number of patients in the studies.

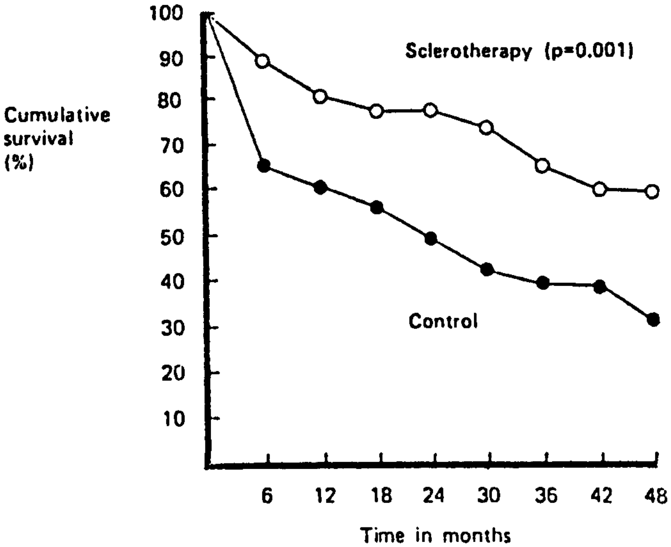

**Figure 4** Cumulative survival curve of the controlled trial at Kings College Hospital in London comparing elective endoscopic sclerotherapy and control; bleeding in the control group was not usually managed by endoscopic sclerotherapy[4].

**Table 6** Estimated natural history after variceal bleeding in patients with liver cirrhosis according to CHILD's classification [from GRAHAM and LACEY-SMITH[10] and BURROUGHS *et al.*[11]]

| *Child classification* | *Survival rate after* 1 month | 1 year | 2 years |
|---|---|---|---|
| A | 85 | 76 | 65 |
| B | 75 | 52 | 39 |
| C | 65 | 35 | 23 |

**Table 7** Survival following prospective paravariceal injection sclerotherapy of 200 consecutive patients according to CHILD's classification[11]

| *Classification* | *No. of patients* | *Survival* 1 year | 2 years |
|---|---|---|---|
| Child A | 45 | 99 | 95 |
| Child B | 60 | 79 | 78 |
| Child C | 95 | 62 | 53 |

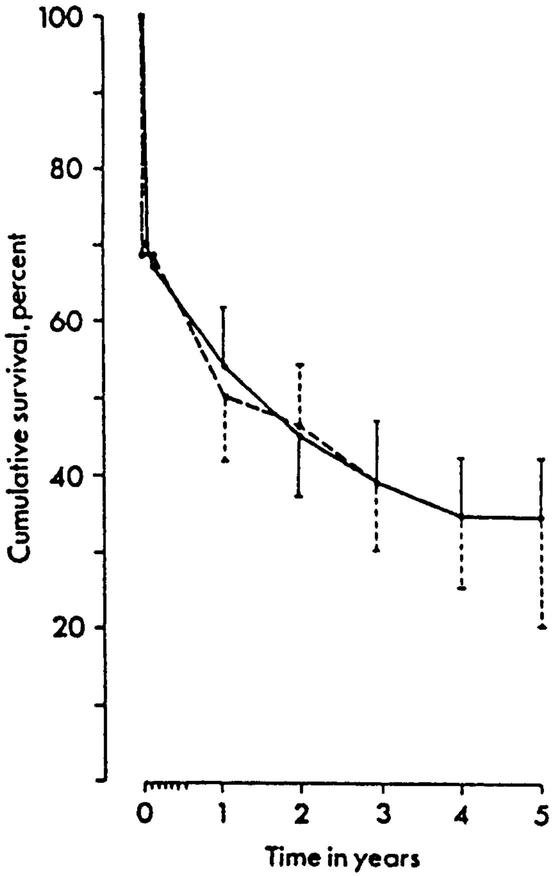

**Figure 5** Cumulative survival in the controlled trial of TERBLANCHE *et al.*[5] comparing elective endoscopic sclerotherapy (———) and controls (- - - - - -); bleeding and controls were managed by emergency endoscopic sclerotherapy.

**Table 8** Rebleeding and survival in controlled randomized trial comparing sclerotherapy (ES) to shunt operation (SO)

| *Author* | *shunt* | *No. of patients* ES | SO | *Rebleeding* ES % | SO | *Time of two years survival* ES % | SO |
|---|---|---|---|---|---|---|---|
| Warren *et al.* 1986 | spleno-renal shunt | 36 | 35 | 53 | 3 | 84(+31 surgery) | 59 s |
| Rikkers *et al.* 1987 | spleno-renal | 30 | 27 | 57 | 19 | 61 | 65 ns |
| Cello *et al.* 1987 | porto-caval (CHILD C) | 32 | 32 | 41 | 16 | 50 | 44 ns |
| Teres *et al.* 1987 | "so-called" spleno-renal shunt | 55 | 57 | 32.5 | 14.3 | 68 | 71 ns |

trial reviewed improved survival under long-term sclerotherapy after 40 days of the initial bleeding episodes[7]. Our group has compared the estimated natural history of variceal bleeding in patients with cirrhosis according to CHILD-PUGH classification, as shown in Table 6 with the life expectancy of 200 consecutive cirrhotics with bleeding esophageal varices treated by endoscopic sclerotherapy prospectively[9]. By this treatment option life expectancy could be prolonged in CHILD A-patients from 65 to 95%, in CHILD B-patients from 39 to 78% and in CHILD C-patients from 23 to 53% (Table 8).

However, rebleeding during initial and long-term injection sclerotherapy occurs in 23 to 55% and remains a problem, even if it is of minor intensity. Therefore, a surgical approach to the so-called "sclerotherapy failures" has been discussed and recently investigated in controlled randomized trials. These randomized studies showed contradictory results (Table 8): recurrence of haemorrhage could be significantly prevented by elective or urgent shunt operation, but survival was only improved in one study, when sclerotherapy failure were backed up by early surgical treatment (Figure 6).

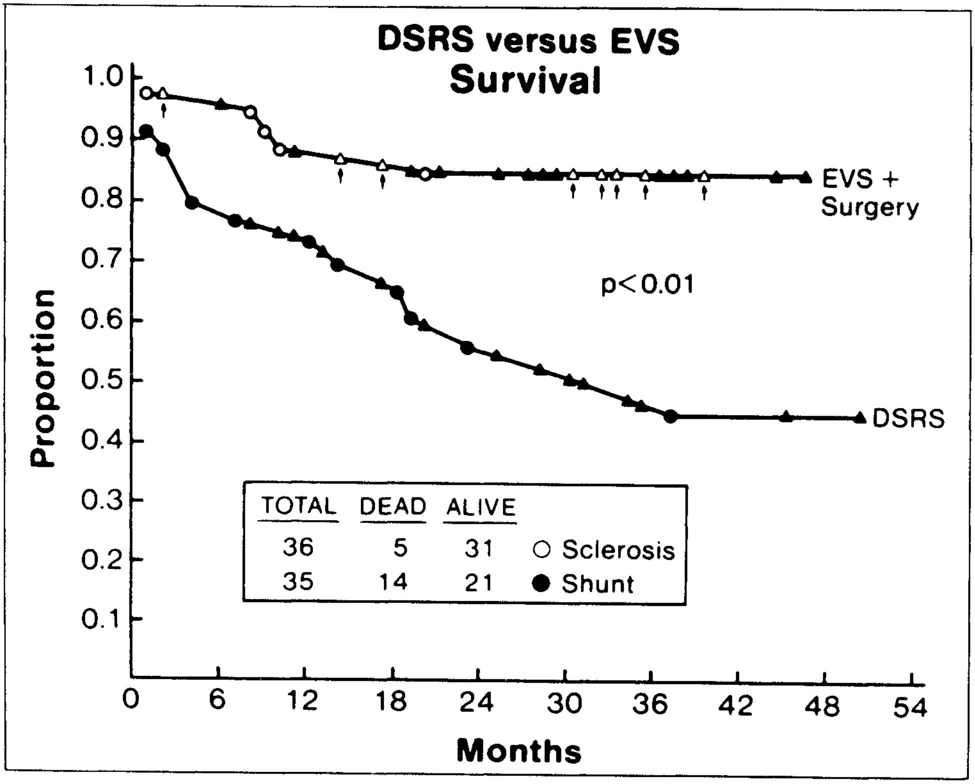

**Figure 6** Preliminary results of the controlled trial of the WARREN-group comparing elective endoscopic sclerotherapy to elective distal splenorenal shunt operation; about 30% of the sclerotherapy group moved to the shunt group because of recurrences of haemorrhage and were declared as "failure".

Since 1975 our group has practised a specific surgical approach in the "long-term sclerotherapy failures". This strategy is based on a definition of sclerotherapy failures and special selection of patients for elective shunt procedures. "Sclerotherapy failure" is defined as either at least two early or late recurrences of oesophageal variceal bleeding during the course of endoscopic sclerotherapy, one recurrent haemorrhage from gastric varices or recurrent bleeding from esophageal ulcers as a sequelae of balloon tamponade and/or endoscopic sclerotherapy.

### *Surgical Strategy and Selection*

In patients belonging to CHILD-PUGH classification C with uncontrollable recurrent variceal haemorrhage an emergency gastroesophageal disconnection (with or without splenectomy or fundoplication) is performed according to HASSAB-PAQUET (Figure 7). In a few cases with portal pressure over 40cm $H_2O$ an emergent or urgent narrow lumen meso-caval shunt is carried out.

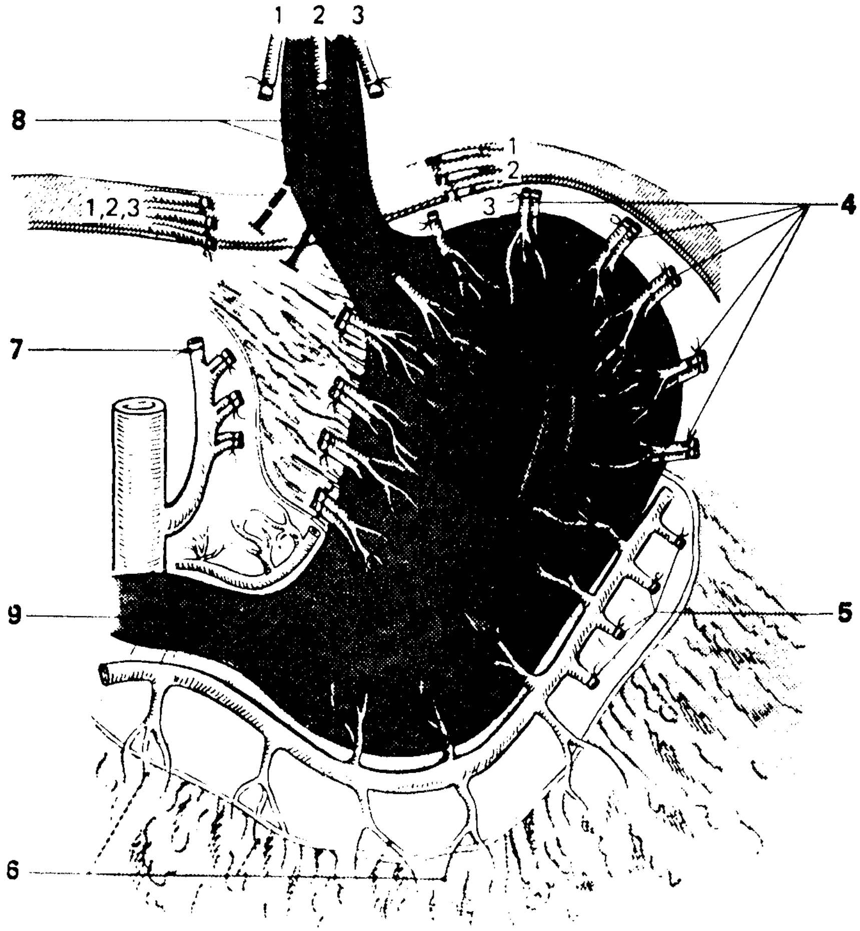

**Figure 7** Gastro-oesophageal disconnection according to HASSAB-PAQUET: devascularisation of the upper two thirds of the stomach and 6cm of the abdominal oesophagus, separation of all connections to the retroperitoneum and the diaphragm and selective proximal vagotomy.

CHILD-PUGH A- and -B-patients are considered for an elective shunt operation according to the criteria listed in Table 10: sonographic volume of the liver should be between 1000 and 2500ml. Portal perfusion rate is measured by sequential scintigraphy; if it is more than 30%, the patient is considered for distal splenorenal shunt. If portal perfusion rate is between 10 and 30%, the patient is considered for a non-selective shunt. The preferred non-selective shunt is the narrow lumen meso-caval interposition shunt (NLMS: 10-12mm ring enforced PTFE-prothesis). Coeliac angiography should exclude stenosis of the hepatic artery and/or coeliac axis as well as "portal pseudoperfusion". Liver biopsy performed at laparoscopy should not show activity of the liver disease.

**Table 9** Selection criteria for elective narrow lumen mesocaval shunt interposition (NLMS) or a distal splenorenal shunt (DSRS) in cirrhotics with recurrence of haemorrhage from oesophago-gastric varices

1. Liver volume, sonographically determined as 1000–2500 ml
2. Portal perfusion 10–30% at sequential scintigraphy
3. No activity or progression of cirrhosis seen at laparoscopy and biopsy
4. No stenosis of the hepatic arterial circulation, and suitable lumen and length of the splenic vein, found at angiographic studies

**Table 10** Number and classification of 692 consecutive patients admitted to the HEINZ-KALK-Hospital because of bleeding from oesophagogastric varices and modality of therapy (Jan. 1, 1982 - Jan. 1, 1989)

| | |
|---|---|
| *692 patients* | |
| 14 | exclusions (2%) |
| 311 | CHILD-PUGH C (45%) long-term endoscopic sclerotherapy |
| 26 | emergent gastroesophageal disconnection according to HASSAB-PAQUET = 8.5% |
| 5 | emergent narrow-lumen mesocaval interposition shunts (1.5%) |
| 367 | CHILD-PUGH A + B |
| 181 | CHILD-PUGH A (26%) |
| 185 | CHILD-PUGH B (27%) |

### MATERIAL AND RESULTS

From January 1, 1982 to January 1, 1989, 692 consecutive patients were admitted to the HEINZ-KALK-Hospital with bleeding oesophageal varices (Table 10). Fourteen patients had to be excluded from the prospective evaluation because of uncontrollable haemorrhage during the first 12 to 24 hrs, or refusal of treatment. In 311 CHILD-PUGH C-patients long-term injection sclerotherapy was performed; 26 of them needed an emergency gastroesophageal disconnection because of uncontrollable or early recurrent haemorrhage, and in 5 an NLMS. In the remaining 367 patients — 182 of them were CHILD-PUGH A and 185 B – endoscopic scleratherapy was successful in 194. In 173 patients, with at least two rebleedings despite long term sclerotherapy, specific selection criteria were used to assess suitability for shunt. Eighty five patients refused shunt operation or did not fulfill selection criteria: in this group endoscopic sclerotherapy was continued. Eighty-eight patients were shunted (Table 11): 54 narrow lumen mesocaval 32 distal spleno-renal shunts, 1 porto-caval and one spleno-renal LINTON-shunt. The continued sclerotherapy and shunt groups were comparable concerning number, demographic characteristics, aetiology, severity and histology of liver disease (Table 12). There was no significant difference of mortality at 30 days (5 vs. 7%). Twenty nine patients from the surviving 81 receiving continued sclerotherapy (36%) died during the later follow up. Mean follow-up time in both groups was 43 months. Four patients of endoscopic sclerotherapy and 3 of the shunt-group were lost to follow-up after 18 to 39 months. Seventeen shunt-patients died during the late follow-up (18%). The cumulative survival curves, calculated using the method of KAPLAN-MEIER are shown in Figure 8. The two curves are significantly ($p < 0.01$) different in favour of patients selected for shunt after sclerotherapy had failed.

**Table 11** Modalities of therapy in 367 patients with CHILD-PUGH A- and -B-classification and types of shunts performed

| | |
|---|---|
| *367 patients (CHILD-PUGH A + B)* | |
| 194 | long-term endoscopic injection sclerotherapy |
| 173 | patients with at least two rebleedings despite effective long-term endoscopic sclerotherapy |
| 85 | patients (shunt refused (69) or selection criteria not fullfilled (26)) |
| 88 | patients were shunted |
| 54 | narrow-lumen meso-caval interposition shunt (NLMS) |
| 32 | distal spleno-renal shunt (DSRS) |
| 1 | porto-caval shunt |
| 1 | proximal spleno-renal shunt (LINTON) |

**Table 12** Demographic characteristics, aetiology, severity and histology of the liver disease, early and long-term results in 173 patients, either selected for endoscopic sclerotherapy or shunt operation

| | *Endoscopic sclerosis* | *Shunt operation* |
|---|---|---|
| Number of patients | 85 | 88 |
| CHILD-PUGH A | 41 (48%) | 40 (46%) |
| CHILD-PUGH B | 44 (52%) | 48 (55%) |
| alcoholic cirrhosis | 57 (67%) | 57 (65%) |
| posthepatitic cirrhosis | 19 (22%) | 20 (22%) |
| other types of cirrhosis | 9 (11%) | 11 (13%) |
| mortality at 30 days | 4 (5%) | 6 (7%) |
| late mortality (up to Jan. 1, 1989) ($p<0.01$) | 29 (36%) | 14 (17%) |

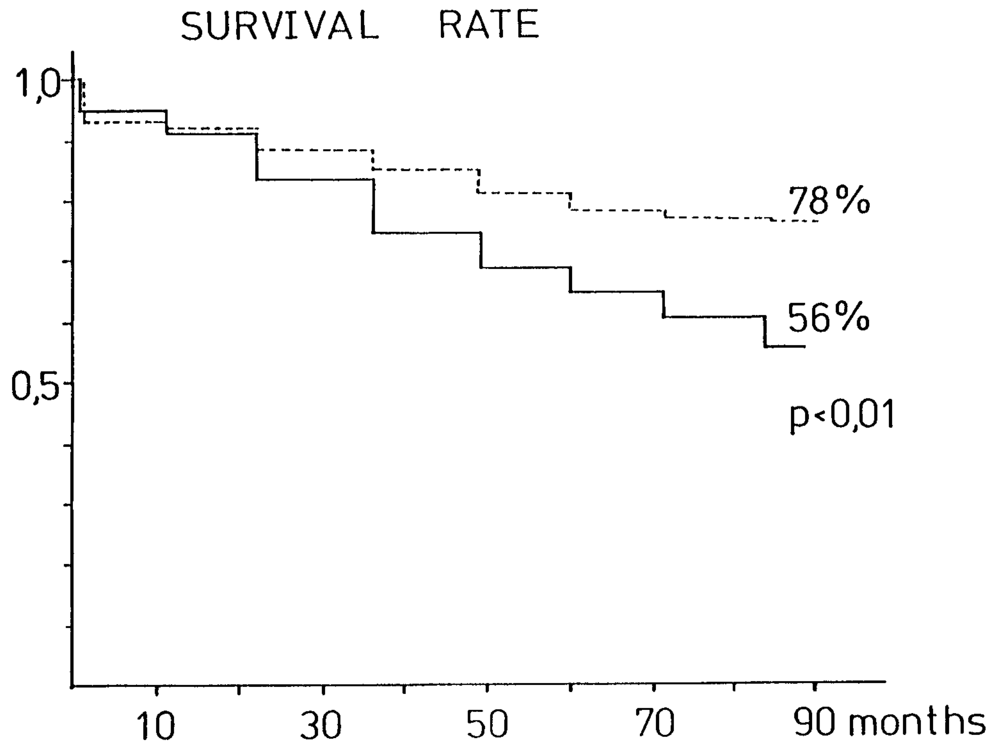

**Figure 8** Cumulative survival curve according KAPLAN-MEIER comparing elective endoscopic sclerotherapy to elective narrow-lumen mesocaval or distal splenorenal shunt in a prospective study (Broken line = shunt).

### *Conclusions*

The art of managing patients with current variceal bleeding during continued endoscopic sclerotherapy is to balance the risk of hepatic failure and recurrent bleeding. Today endoscopic sclerotherapy is the best first line of treatment, and is more effective than conservative therapy alone, in preventing rebleeding and improving survival. Endoscopic sclerotherapy does not change total liver perfusion, portal perfusion and thus liver function. On the other hand, in spite of long-term and effective sclerotherapy at least 30% of early and late rebleeders should be expected, with the risk of liver failure induced by rebleeding. Acute rebleeders are best managed by devascularisation procedures and rarely by emergency shunt operations in spite of the fact that it is questioned that this strategy instead of chronic sclerotherapy can improve survival. Those patients who rebleed need urgent and effective therapy. In chronic rebleeders selective distal splenorenal shunt and non-selective narrow-lumen meso-caval shunts can prevent early and late rebleeding without negative influence on liver haemodynamics and hepatic function in CHILD-PUGH A- and -B-patients. Furthermore these shunts are able to prolong survival without deteriation of liver function or a higher frequency of liver failure on long term basis. Our trial demonstrates for the first time that this is particularly true for CHILD-PUGH A- and -B-patients who fulfill special selection criteria. Furthermore non-selective narrow lumen meso-caval interposition shunt has been proved to be a good alternative to selective distal splenorenal Warren-shunt if this is technically impossible or haemodynamically not advisable.

### *References*

1. Paquet, K-J. and Feussner, H. (1985) Endoscopic sclerosis and esophageal balloon tamponade in acute haemorrhage from esophagogastric varices: a prospective controlled randomized trial. *Hepatology* **5**, 580–583
2. Larson, A.W., Cohen, H, Zweiban, B., Chapman, D., Gourdji, M., Korula, J. and Weiner, J. (1986) Acute esophageal variceal sclerotherapy. Results of a prospective randomized controlled trial. *Journal of the American Medical Association* **53**, 457–481
3. Paquet, K-J., Kalk, J-Fr. and Koussouris, P. (1988) Immediate endoscopic sclerosis of bleeding esophageal varices - a prospective evaluation over five years. *Surg. Endoscopy* **2**, 18–23
4. MacDougall, B.R.D., Westaby, D., Thodossi, A. *et al* (1982) Increased long-term survival in variceal haemorrhage using injection sclerotherapy. Results of a controlled trial. *Lancet* **1**, 124–127
5. Terblanche, J., Bornman, P.C., Kahn, D. *et al.* (1983) Failure of repeated injection sclerotherapy to improve long-term survival after esophageal variceal bleeding. A five year prospective controlled clinical trial. *Lancet*, **2**, 1328–1332
6. Soederlund, C. (1985) Endoscopic sclerotherapy of esophageal varices, a clinical study. *Acta Chirurgica Scandanavica Supplement* **151**, 1–23
7. The Copenhagen Variceal Sclerotherapy Project (1984) Sclerotherapy after first variceal haemorrhage in cirrhosis, a randomized multicenter trial. *New England Journal of Medicine* **311**, 1594–1600
8. Korula, J., Balart, L.A., Radvan, G., Zweiban, B.E., Larson, A.W., Kao, H.W. and Yamada, S. (1985) A prospective randomized multicenter trial of chronic esophageal variceal sclerotherapy. *Hepatology* **5**, 584–589
9. Paquet, K-J. (1988) Indications and early and long-term results of paravariceal immediate, elective and prophylactic injection sclerotherapy. In: Y. Idezuki: Treatment of esophageal varices Excerpta Medica, pp. 1–22, Amsterdam -New York - Oxford
10. Graham, D.Y., Lacey-Smith, J. (1981) The course of patients after variceal haemorrhage. *Gastroenterology* **80**, 800–809
11. Burroughs, A.K., Sanchez, A., Bass, N.M. *et al.* (1983) Can endoscopic sclerotherapy influence significantly the course of cirrhotic patients who survive variceal bleeding? *Gut* **24**, 972–977
12. Warren, W.D., Henderson, J.M., Millikan, W.J. *et al.* (1986) Distal splenorenal shunt vs. endoscopic sclerotherapy for long-term mamangement of variceal bleeding: a report of a prospective randomized trial. *Annals of Surgery* **203**, 454–462
13. Rikkers, L.F., Burnett, D.A., Volentine, G.D., Buchi, K.N. and Cornier, R.A. (1986) Shunt surgery vs. endoscopic sclerotherapy for long-term management of variceal bleeding. Preliminary report of a prospective randomized trial. *Annals of Surgery* **203**, 454–462
14. Terés, J., Bordas, J.M., Bravo, D., Wisa, J., Grande, L., Garcia-Valdecasas, J.C., Pera, C.H. and Rodés J. (1987) Sclerotherapy versus distal splenorenal shunt in the elective treatment of variceal haemorrhage: a randomized controlled trial. *Hepatology* **7**, 430--436
15. Cello, J.P., Grendel, J.H., Crass, R.A., Weber, T.H.E., Trunkey, D.D. (1987) Endoscopic sclerotherapy versus portocaval shunt in patients with severe cirrhosis and acute variceal haemorrhage - long term follow up. *New England Journal of Medicine* **316**, 11–15
16. Paquet, K-J., Mercado, M.A., Koussouris, P., Kalk, J-Fr., Siemens, F. and Cuan-Orozco, F. (1989) Improved results with selective distal splenorenal shunt in a highly selected patients population - A prospective study. *Ann. Surg.* **210**, 184–189
17. Paquet, K-J., Mercado, M.A., Kalk, J-Fr., Koussouris, P., Siemens, F. and Müting, D. (1990) Analysis of a prospective series of 100 mesocaval interposition shunts for bleeding portal hypertension. *Hepato-gastroenterology* **37**, 115–120

18. Paquet, K-J., Mercado, M.A. Gad, H.A. and Cuan-Orozco, F. (1990) Surgical procedures in bleeding esophagogastric varices when sclerotherapy fails – a strategy and prospective study. *American Journal of Surgery* **160**, 43–47

